# The Effects on Inappropriate Weight for Gestational Age of an SMS Based Educational Intervention for Pregnant Women in Xi’an China: A Quasi-Randomized Controlled Trial

**DOI:** 10.3390/ijerph17051482

**Published:** 2020-02-25

**Authors:** Zhongliang Zhou, Yanfang Su, Jesse Heitner, Yafei Si, Dan Wang, Zhiying Zhou, Changzheng Yuan

**Affiliations:** 1 School of Public Policy and Administration, Xi’an Jiaotong University, Xi’an 710049, Shaanxi, China; zzliang1981@xjtu.edu.cn (Z.Z.); dwang1220@163.com (D.W.); 2 School of Medicine, University of Washington, Seattle, WA 98195, USA; yfsu@uw.edu; 3 Aceso Global, Washington, DC 20036, USA; 4 School of Risk and Actuarial Studies and CEPAR, University of New South Wales, Sydney, NSW 2052, Australia; hanshi8860@126.com; 5 School of Public Health, Xi’an Jiaotong University Health Science Center, Xi’an 710061, Shaanxi, China; zzying1982@163.com; 6 The Children’s Hospital and School of Public Health, Zhejiang University School of Medicine, Hangzhou 310058, Zhejiang, China; 7 Nutrition Department, Harvard T.H. Chan School of Public Health, Boston, MA 02115, USA

**Keywords:** inappropriate weight for gestational age, short message service, quasi-randomized controlled trial, mhealth

## Abstract

Background: The aim of this study was to estimate the effects of maternal text messages on inappropriate weight for gestational age (IWGA) in newborns in rural China. Methods: Participants were pregnant women presenting for antenatal care at a Maternal and Child Health Center in Xi’an, China during the 2013–2015 period. In total, 2115 women completed the program with follow-up information included in the final analyses. All mothers were divided into four groups, including (1) a control group that received only a few “Basic” messages, (2) a Care-Seeking (CS) message group, (3) Good Household Prenatal Practices (GHPP) message group, and (4) a group receiving all 148 text messages. The primary outcome was IWGA, including small for gestational age (SGA) and macrosomia (weighing ≥4000g at birth). Multivariable logistic regression using an intent-to-treat estimate was utilized. Results: In total, 19.5% of newborns were IWGA. The risk of IWGA was 23.0% in the control group, 19.6% in the CS group, 18.9% in the GHPP group, and 16.5% in the group with All Texts. Compared to the control group, the odds ratio of IWGA was 0.65 (0.48–0.89) for the group receiving All Texts, which remained statistically significant after performing the Holm-Bonferroni correction. The odds ratio of macrosomia was 0.54 (0.34–0.87) and 0.57 (0.36–0.49) for the Care Seeking message group and the All Texts group, respectively, with statistical significance. Conclusion: A package of free informational text messages, including advice for good household prenatal practices and care seeking, may prevent the inappropriate weight for gestational age through a protective effect on macrosomia. Advice to encourage care seeking in pregnancy may prevent macrosomia among neonates in rural China as well.

## 1. Introduction

The inappropriateness of weight for gestational age (IWGA), used to measure neonatal health, is defined as being either small for gestational age (SGA) or having macrosomia (weighing ≥4000g at birth). By accounting for gestational age, SGA is a commonly accepted proxy for intrauterine growth restriction specifically [1]. A recent meta-analysis pooling 20 cohorts from low- and middle-income countries found that SGA was associated with a relative risk of 1.83 (95% CI: 1.34–2.50) for neonatal mortality and 1.90 (1.32–2.73) for post neonatal mortality [2]. On the other side of the spectrum, excessive fetal growth presents risks as well. A 2013 systematic review and meta-analysis found that macrosomia, neonates weighing ≥4000g, were at elevated risk of shoulder dystocia, birth trauma, asphyxia, and Neonatal Intensive Care Unit admission, though not of perinatal death [3]. Birth trauma in particular seems to increase sharply with the gradation of macrosomia (≥4000g, ≥4500g, ≥5000g) [3,4]. In terms of the maternal and offspring health, macrosomia of the fetus is associated with increased odds of emergency cesarean sections, excessive bleeding, prolonged or dysfunctional labor, and child obesity in the later years of life [4,5,6,7].

As expectant mothers in low- and middle-income countries often lack access to vital information about pregnancy, preparation for birth, and best practices when caring for their newborn, the incidence of IWGA in these countries is relatively high. In 2010, 27% of all live births were born SGA in low- and middle-income countries; meanwhile, the total prevalence of macrosomia was 8.7% in China [8,9]. One potential solution is the use of mobile health (mHealth), and specifically the use of short message service (SMS), to accurately inform underserved women about best practices. In a 2016 review of mobile technology used in the health sector, researchers at Johns Hopkins found that among the disease groups addressed by mHealth interventions, maternal and child health were among the most salient, collectively accounting for about 44% of all programs globally [10]. However, there is limited quantitative research on the effect of SMS interventions via cell phone on maternal and child health outcomes [11,12,13]. Recent systematic reviews assessing the effect of mHealth interventions on improving maternal and neonatal care and health discussed more than ten intervention studies in this field and showed that mHealth interventions targeting pregnant women can increase maternal and neonatal service utilization [14,15,16]. Only one study showed an improvement in morbidity or mortality, specifically decreased risk of perinatal death in children of mothers who received SMS support during pregnancy, compared with routine prenatal care. [17] Otherwise, the various studies in developing countries that have attempted to evaluate the impact of mHealth interventions on maternal and child health have been either inconclusive, or of a very small scale. In summary, the impact of SMS on maternal and child health has not been evaluated in a sufficiently powered randomized or quasi-randomized controlled trial [15].

To address this issue, we designed and implemented a quasi-randomized controlled trial, the Newborn Health Project in rural China, to determine if and how an innovative SMS intervention providing educational information via texts to pregnant women in China might lead to maternal and child health outcomes. A more in-depth discussion of the literature and the study protocol has been reported previously [18]. Our study will add to the literature that maternal health education via cell phone text messages could greatly prevent the occurrence of inappropriate weight for gestational age, particularly on the macrosomic end of the spectrum.

The Newborn Health Project offers expectant mothers in the rural district of Gaoling in Xi’an, China a package of free, short, informational messages via cell phone regarding pregnancy and childbirth. These messages are delivered throughout the pregnancy and are tailored to each mother’s gestational week. The message sending was terminated if the participant sent a “stop” message to our platform. The first version of the text message bank was developed based on an education package donated by Apricot Forest, Inc, for academic use, in 2011. With additional literature review on maternal health education, we ultimately designed 148 messages in 2012. The topics ranged from prenatal lifestyles (e.g., nutrition, prenatal smoking and drinking, etc.), fetus developmental stage, neglected issues (e.g., postpartum depression, pain management practice, etc.), suboptimal practices (e.g., caesarean delivery, breast feeding, etc.), and medical signs for seeking clinical care.

We hypothesized that our SMS intervention might reduce IWGA via several pathways. For example, messages with nutritional advice during pregnancy might reduce the incidence of SGA. Meanwhile, because rural China households might have a preference for large babies, we delivered information about appropriate fetal development, as well as regular moderate exercise, either of which might reduce macrosomia. Promotion of regular antenatal care checkups and appropriate care-seeking during symptomatic illness could potentially result in the early detection and correction of SGA or macrosomia.

## 2. Methods

### 2.1. Study Site

We selected Gaoling County in Shaanxi Province, China. According to County Government statistics in 2013, Gaoling County had a population of 330.000, with 3441 pregnant women in 2012. In addition, 98% of the households in Gaoling owned a cell phone.

### 2.2. Pre-Trial Preparation

This study was approved by the Ethics Committee of the School of Medicine at Xi’an Jiaotong University on 18 January 2013 and an updated version was approved on May 2016 (Approval number: 2016–392). In addition, this study was registered at clinicaltrials.gov (NCT02037087), and the protocol has been published previously [18]. Power calculation to determine sample size was presented in the published protocol [18].

### 2.3. Recruitment & Treatment Assignment

Pregnant women were recruited at Gaoling Maternal and Child Health Center when they presented for antenatal care checkups. All pregnant women at any gestational age who had access to a cellular phone were eligible for our trial. Recruitment began in 2013 and finished in 2015. Upon enrollment, an informed consent form was completed, followed by a baseline survey of demographic, health, and pregnancy related items.

Our study then used a quasi-randomized factorial assignment and placed each participating pregnant woman into one of four possible message package programs. Quasi-randomization assigned treatment based on the expecting mother’s birthday, specifically whether their birth month and day of birth were odd-odd, odd-even, even-odd, or even-even. The four study groups included: (1) a limited set of “Basic” messages about pregnancy, which acts as a control group (25 messages); (2) Care-Seeking (CS) messages, which include information about government-subsidized programs, warning signs of potential problems and the importance of care-seeking during illness (82 messages); (3) Good Household Prenatal Practices (GHPP) messages, including advice on nutrition, exercise, self- awareness of depression, breast feeding, etc. (91 messages); and (4) All Texts messages, which is the full message bank (148 messages). These “Basic” messages in the control group include updates on fetal development in different gestational stages, reminders for prenatal visits and promotion of certified skilled attendance during labor. For comparability, all groups receive these Basic messages. The rationale to distinguish two types of messages is based upon dependence of health systems capacities. More specifically, behaviors encouraged by Care-Seeking messages interact with health systems; while behaviors encouraged by Good Household Prenatal Practices messages are more independent from health systems. Our design enables us to understand maternal behaviors in the household and beyond. From a policy perspective, it is important to disentangle which component contributes most to neonatal health. Therefore, two-by-two intervention assignments was conducted to comparatively evaluate three groups of SMS messages, i.e., GHPP messages, CS messages, and the combination of both types of messaging. A summary of the intervention texts by group is presented in Table 1 and the detailed information can be found in the study protocol published previously [18].

### 2.4. Sample Description

Our program enrolled a total of 4624 women, with 1110, 1173, 1097, and 1244 enrolled in the Basic group, CS group, GHPP group, and All Texts group, respectively. Here in Table 2 we present a description of summary statistics of the measured baseline characteristics of the sample.

### 2.5. Outcome Classification

Newborn birth weight was self-reported by new mothers to health workers in the follow-up survey in the first home visit after delivery. All participants in the study had facility deliveries in the local Maternal and Child Health Center or hospitals, and newborn birth weight was measured by health professional right after delivery. Within one week after delivery, local health workers collected health information in a home visit for all new mothers, regardless of differences in delivery locations. For our primary outcome, IWGA was defined as being either small for gestational age (SGA) or having macrosomia. SGA was defined as birthweight below that predicted for the 10th percentile for gestational week, which was predicted by Mikolajczyk et al.’s formula [19], in accordance with the WHO’s (World Health Organization) standard definition of SGA [1]. Macrosomia was defined as weighing ≥4000 g at birth.

### 2.6. Statistical Analyses

To analyze the effectiveness of the different treatment groups using intent-to-treat (ITT), unadjusted and multivariable logistic regressions were applied. Though our balance check showed indicated that our randomization was successful at balancing observable covariates, as a robustness check we performed a subsequent regression in which we included all covariates that were found to be unbalanced at *p* < 0.10 for the full sample at baseline as well as those baseline covariates which a balance check limited to the 2115 participants completing the study found unbalanced at *p* < 0.10. Included covariates were gestational week at enrollment, history of miscarriage, the smoking status of the woman and her husband, the woman’s self-rated health compared to before pregnancy, the woman’s gender preference for her child, and self-reported intentions to be proactive about health during pregnancy. As a second robustness check, a logistic regression was performed which contained all unbalanced baseline covariates as the regression above as well as an array of general health, socioeconomic and maternal which might influence or predict a newborns status as IWGA. General health variables included the mother’s pre-pregnancy body mass index (BMI) and exercise habits at baseline. Socioeconomic variables included income, residence, education, and insurance coverage (medical insurance for rural residents). Maternal indicators included whether the mother previously had a live birth, whether the pregnancy was planned, and family gender preferences.

As defined, inappropriate weight for gestational age is composed of two components; small for gestational age (SGA) and macrosomia. These components comprise opposite ends of the weight spectrum, and our measured effects could result from a change to either or both poles. Further, one possibility is that a reduction in incidence of one measure could be offset by an increase in incidence of the other. To investigate the source of our intervention’s measured effects, we further decomposed our fully adjusted logistic regression into two: one on SGA and one on Macrosomia.

## 3. Results

In total, 2115 women of the original 4624 from baseline completed a post-delivery follow-up survey. Of this, 526 (24.9%) were in the control group, 518 (24.5%) were in the CS group, 497 (23.5%) were in the GHHP group, and 574 (27.1%) were in the group receiving All Texts. A chi-squared test shows no evidence of differential loss to follow-up by treatment group, failing to reject the null of equal attrition at *p* = 0.435. The study’s overall loss to follow-up rate was 55.3%.

In total, 243 (11.6%) of newborns were SGA. In terms of macrosomia, 164 newborns (7.8%) were born macrosomic. In total, 407 (19.5%) of newborns were an inappropriate weight for their gestational age. The number of newborns with IWGA was 120 (23.0%) in the control group, 100 (19.6%) in the CS group, 93 (18.9%) in the GHPP group, and 94 (16.5%) in the group with All texts.

In the unadjusted analyses, compared to the control group which received only a small amount of basic messages, the ORs (95% CIs) of delivering their newborn with an inappropriate weight for their gestational age (IWGA) were 0.80 (0.60–1.08) for the group receiving Care Seeking messages and 0.80 (0.59–1.08) for the group receiving Good Household Practices messages, without statistical significance (see Model 1 in Table 3). The corresponding ORs (95% CIs) for the group receiving all program texts was 0.66 (95% CI: 0.49–0.89; *p* < 0.01). This remained statistically significant after performing the pre-planned Holm-Bonferroni correction for 4 group comparisons, and even after performing a more conservative 6-test correction for all possible group comparisons between four treatment groups.

After further adjustment of gestational week at enrollment, history of miscarriage, the smoking status of the woman and her husband, the woman’s self-rated health compared to before pregnancy, gender preference for her child, and the self-reported intentions to be proactive about health during pregnancy, the estimated ORs (95% CI) remained basically the same for all pairwise comparisons (see model 2 in Table 3). Moreover, adding additional covariates including general health variables, socioeconomic variables and maternal indicators in the multivariable model again had negligible effect on regression results for our treatment group variables. The effect measures were slightly stronger, and the statistical significance remained unaltered (see Model 3 in Table 3).

When decomposing our analyses into SGA and macrosomia, we didn’t observe a statistically measurable effect of treatment on SGA, though the All Texts group had a fairly large but statistically insignificant effect measure of 0.76 (95% CI: 0.52–1.13) (Table 4). With regards to macrosomia, both the Care Seeking message group and the All Texts group had significantly lower odds of delivering macrosomic newborns compared to the control group. The corresponding ORs (95% CI) were 0.54 (0.34–0.87) and 0.57 (0.36–0.49), respectively.

## 4. Discussion

In our study population, we observed a rate of 11.6% for SGA in Xi’an. This is close to, but slightly higher than the 10% that would be expected for China as a nation under the formula created by Mikolajczyk et al. [19], which was used in this study to generate our reference standard for China’s national weight for age distribution [19]. The definition of SGA is meant to encompass the lowest decile of weight for gestational age, and it may be that newborn weight in Xi’an is slightly lower than China’s national average predicted by Mikolajczyk et al.’s formula. In terms of macrosomia, 7.8% were born macrosomic in Xi’an, which is quite close to Lu et al.’s national estimate of 7.83% [20].

In this large, quasi-randomized educational trial among 2115 pregnant women in rural China compared to the group which received only basic messages, women who received a full package of free informational text messages regarding pregnancy and childbirth had a lower odds of delivering their newborn with inappropriate weight for gestational age. The effect on birth weight was more pounced for macrosomia, when both Care Seeking message and the full program messages had much lower odds of delivering macrosomia newborns compared to the control group. The relation remained consistent in a large number of robustness check analyses. Overall, this study strongly suggests that our study educational text messages can potentially reduce the odds that neonates are born with an inappropriate weight for their gestational age in rural China.

This study is the first large scale international study quantifying the impact of health text messages via cell phone on birth weight in rural China. Another interesting finding in our study is that receiving all program texts reduces IWGA through a protective effect on macrosomia, while no statistically significant effect on SGA was observed. While further investigation is warranted to elucidate this observation, there may be an additional and smaller protective effect against SGA. Reducing the incidence of the former whilst also possibly protecting against the latter suggests the full text message bank was successful in inspiring a Pareto improvement in gestational weight, rather than a shift that traded overweight for underweight.

While evidence is still accumulating, the observed impact on birth weight may be explained by several pathways. In our study, the total message bank contained 148 messages delivered throughout pregnancy, including but not limited to exercise, micronutrient supplementation, or care-seeking. Moderate exercise has generally been viewed as an avenue to potentially preclude the opposite problem of Large for Gestational Age as well as to guard against gestational diabetes. The relationship between moderate exercise and low birth weight or SGA is an active area of research. Potential concern has been raised as to whether moderate exercise actually may contribute to SGA for pregnancies at-risk for the condition. However, both a 2011 review of 35 relevant studies and a 2015 meta-analysis of 79 studies conclude that prenatal exercise seems to protect against “large at birth,” defined as either Large for Gestational Age or having macrosomia, without any increase in the odds of “small-at-birth,” defined as either SGA or low birth weight [21,22]. There is also a recent trend in findings suggesting that regular low to moderate exercise reduces the risk of delivering the newborns with either very low or very high birth weight [23], though this potential benefit was not detected in Wiebe et al.’s 2015 meta-analysis of 79 studies. In terms of maternal nutritional supplementation, evidence suggesting a possible impact on low birth weight was limited for antenatal iron supplementation and mixed for multi-micronutrient supplementation [24,25,26]. Moreover, messages of advice on other topics may potentially have played a role, such as advice on growth monitoring, anxiety reduction, or others.

This current study has multiple strengths. The quasi-randomized study design, large sample size, and detailed recording of health outcomes allowed us to quantitatively examine the effect of maternal text message health education on neonatal birth weight and to provide precise effect estimates. The selection of inappropriate weight for gestational age as our primary health outcome took into consideration of both the low end and the high end of birth weight. In addition, to categorize infants as SGA, we utilized the formula refined and validated by Mikolajczyk et al. [19]. The authors validated their standard using the data from the 24 countries in the WHO Global Survey on Maternal and Perinatal Health, and found it to be a better predictor of adverse outcomes for neonates than non-customized standards [19]. 

The major limitation of our study is the recall of birth weight. However, this was likely to be reasonably accurate since recalling was done within one week after the delivery, but may have induced some measurement error. Our study is also limited by the study population being concentrated in one county of one province; it is unknown whether the results would remain the same in other counties and other provinces. Additionally, very high loss to follow-up lowered the planned statistical power of our study. Major reasons for loss to follow up include miscarriage, abortion, changing residency, inaccurate or missing information to match between surveys. Loss to follow-up does not seem associated with treatment assignment, and baseline covariates are balanced within both the full sample and the subset that completed the study, which suggests loss to follow-up is hopefully not biasing our results. However, we cannot measure whether it is associated with outcomes, and in particular with mode of delivery, and as such we cannot confidently rule out the possibility that high loss to follow-up has altered our findings. Finally, despite quasi-random assignment and successful balance checks on observables, imbalance in unobserved covariates could give rise to a spurious correlation between treatment assignment and IWGA.

## 5. Conclusions

With the recent relaxations in China’s one child policy, this number of newborns could grow considerably in the next few years, which translates to over a million SGA births and a million macrosomic births in China each year. The full text message bank of our intervention has been shown in our study area to be associated with a statistically significant decline in IWGA, particularly on the macrosomic end of the spectrum. Though it is unclear by what mechanism this association has arisen, if it holds in the broader population, it is possible that many thousands of IWGA births could be prevented with more widespread dissemination of intervention texts. Replication study in similar Chinese settings is warranted as a precursor for possible scale-up and widespread dissemination.

## Figures and Tables

**Table 1:** Short Message Service (SMS), by General Topic and Treatment Group.

Randomized Group	Message Categories
Basic Group (25)	Fetal development (19)
	Reminders for prenatal visit and hospital delivery (6)
Care-Seeking (CS) Group (82)	Fetal development (19)
	Reminders for prenatal visit and hospital delivery (8)
	Warnings & Recognition of danger signs (45)
	Reminders for government-subsidized projects (10)
Good Household Prenatal Practices (GHPP) Group (91)	Fetal development (19)
	Reminders for prenatal visit and hospital delivery (6)
	Healthy lifestyle (Nutrition, physical activity, etc.) (37)
	Mental health during pregnancy (8)
	Pain management (9)
	Labor (6)
	Breastfeeding (6)
All Texts Group: Full SMS Bank (148)	Full bank (148)

Note: See details about design of test messages on pp 4–6 in published protocol by Su et al. 2016 [18]. doi 10.1136/bmjopen-2015011016.

**Table 2:** Summary Statistics and Balance Check.

	Full Sample	Basic Group	CS Group	GHPP Group	All Texts Group	*p*
Age, years old, mean (sd)	27.5 (3.9)	27.5 (4.0)	27.5 (3.9)	27.4 (3.8)	27.6 (3.9)	0.811
Height, cm, mean (sd)	160.8 (4.7)	160.9 (4.8)	160.9 (4.8)	160.8 (4.7)	160.6 (4.4)	0.289
Weight, Kg, mean (sd)	62.0 (20.0)	61.8 (19.2))	62.4 (20.2)	61.7 (20.3)	62.2 (20.3)	0.807
Ethnicities, %						
Han	99.1	99.4	99.1	99.3	98.7	0.343
Other Ethnicities	0.9	0.6	0.9	0.7	1.3	
Phone Owned or Not, %						
Phone Self-Owned	91.5	91.6	90.8	91.6	92.1	0.684
Use Others’ Phone	8.5	8.4	9.2	8.4	7.9	
Marriage Status, %						
Currently Married	98.5	98.5	98.6	98.6	98.1	0.726
Other Status	1.5	1.5	1.4	1.4	1.9	
Gestational Age at Enrollment, week, mean (sd)	15.1 (7.5)	14.9 (7.3)	15.2 (7.4)	15.2 (7.4)	15.1 (7.7)	0.764
Residency area, %						0.252
Province/City	2.9	2.4	2.7	2.6	3.7	
County	14.8	13.6	15.4	16.2	14.0	
Township	18.5	20.7	17.9	17.7	17.8	
Village	63.9	63.3	64.0	63.5	64.5	
Education Level, %						0.321
Junior High or Less	43.0	45.9	43.5	40.1	42.6	
Senior High / Technical	28.2	27.5	27.2	30.5	27.7	
3year College	21.1	19.7	21.6	21.9	21.3	
4year College +	7.7	6.9	7.7	7.5	8.4	
Insurance, %						0.664
Medical Insurance for Rural Residents	77.4	78.9	77.0	76.3	77.5	
Medical Insurance for Urban Workers	6.8	5.4	7.4	7.2	7.0	
Medical Insurance for Urban Residents	8.7	9.5	8.5	8.5	8.3	
Other Medical Insurance	2.1	2.1	2.2	2.3	1.7	
None	5.0	4.1	4.9	5.7	5.5	
Income, CNY, mean (sd)	58,763.7 (16,108.7)	48,797.8 (47,584.6)	57,044.7 (86,324.6)	74,113.6 (30,802.3)	55,507.4 (52,962.5)	0.317
Number of Pregnancies, %						0.960
1st	43.2	42.9	43.7	43.5	42.7	
2nd	35.1	35.8	35.2	33.9	35.4	
3rd +	21.8	21.3	21.2	22.7	21.9	
Past Live Births or Not, %						
Any Past Live Births	35.6	36.0	34.7	35.7	36.0	0.894
No Past Live Births	64.4	64.0	65.3	64.3	64.0	
Past Miscarriages or Not, %						
Any Past Miscarriages	42.6	41.3	43.6	43.1	42.6	0.729
No Past Miscarriages	57.4	58.7	56.5	56.9	57.4	
Previous Delivery Gender, %						0.917
Female	63.4	64.1	62.6	64.4	62.5	
Male	36.7	35.9	37.4	35.6	37.5	
Previous Birth Preterm, %						0.140
Yes	5.1	7.2	3.7	5.3	4.3	
No	94.9	92.8	96.3	94.7	95.7	
Health Condition Before Pregnancy, %						0.441
Very Good	8.2	8.6	7.7	8.0	8.3	
Good	49.6	47.1	50.1	50.1	50.7	
Fair	40.6	42.6	40.4	40.9	38.9	
Poor / Very Poor	1.6	1.7	1.8	0.9	2.1	
Health Compared to Before Pregnancy, %						0.094
Better	4.4	4.0	4.6	5.3	3.8	
The Same	63.3	62.8	60.9	65.5	64.0	
Worse	20.6	21.7	23.0	17.9	19.6	
Don’t know	11.8	11.6	11.5	11.3	12.6	
Current Smoker, %						0.199
Yes	1.2	1.7	0.8	0.9	1.4	
No	98.8	98.3	99.2	99.1	98.6	
Husband Smoke, %						0.069
Yes	54.7	56.0	56.2	51.3	55.0	
No	39.3	37.7	37.5	43.6	38.6	
Former	6.1	6.3	6.4	5.1	6.3	
Current Drinker, %						0.419
Yes	1.4	1.0	1.8	1.1	1.5	
No	98.6	99.0	98.3	98.9	98.5	
Exerciser, %						0.762
Yes	33.6	34.7	33.1	34.3	32.4	
No	55.5	55.4	55.5	54.3	56.5	
Former	11.0	9.9	11.4	11.5	11.1	
Planned Pregnancy, %						0.523
Yes	65.9	64.2	66.7	67.0	65.9	
No	34.1	35.8	33.3	33.1	34.1	
Family Gender Preference, %						0.499
Boy	8.0	8.3	7.0	7.9	8.8	
Girl	7.7	7.6	7.7	6.6	8.5	
No Preference	84.4	84.2	85.3	85.5	82.7	
Women Gender Preference, %						0.092
Boy	7.6	9.1	6.3	7.1	7.9	
Girl	19.8	21.3	19.3	20.3	18.4	
No Preference	72.7	69.6	74.4	72.6	73.7	
Intentions to be Proactive on Health, %						0.042
No Intention	1.7	1.3	3.0	1.5	1.1	
Few Intentions	4.9	4.3	5.2	4.8	5.2	
Somewhat Intend	36.5	36.9	36.6	36.2	36.4	
Intend	38.5	38.1	37.2	40.8	38.0	
Strongly Intend	15.8	16.4	15.4	15.1	16.1	
Don’t Know	2.6	3.0	2.6	1.6	3.3	

Note: For continuous and ordinal variables, the *p* values were calculated from the one-way ANOVAs, and for categorical variables, the *p* values were calculated from chi-squared tests. Abbreviation: standard deviation (sd).

**Table 3:** Logistic Regression models with inappropriate weight for gestational age (IWGA) as the dependent variable.

Variables	Model 1		Model 2		Model 3	
	Odds Ratio	95% CI	Odds Ratio	95% CI	Odds Ratio	95% CI
Treatment Assignment						
Control Group	Reference		Reference		Reference	
CS Group	0.80	0.60–1.08	0.81	0.60–1.09	0.79	0.58–1.07
GHPP Group	0.80	0.59–1.08	0.78	0.58–1.06	0.77	0.57–1.05
All Texts Group	0.66 ***γ ϕ	0.49–0.89	0.66 ***γ ϕ	0.49–0.89	0.65 ***γ ϕ	0.48–0.89
Gestational Week At Enrollment			1	0.99–1.02	1	0.99–1.02
Past Miscarriage			1	0.80–1.26	1	0.79 1.27
Smoker			0.61	0.17–2.15	0.60	0.17–2.16
Husband Smoker			0.88	0.71–1.10	0.88	0.69–1.11
Health Compared to Before Pregnancy						
Better			0.66	0.37–1.20	0.67	0.37–1.21
The Same			Reference		Reference	
Worse			0.78 *	0.58–1.04	0.78 *	0.57–1.05
Not Sure			0.81	0.56–1.19	0.8	0.54–1.18
Maternal Gender Preference						
No Preference			Reference		Reference	
Prefer Boy			0.96	0.61–1.50	1.11	0.67–1.82
Prefer Girl			1.19	0.90–1.58	1.24	0.91–1.70
Intentions to be Proactive on Health			0.94	0.83–1.06	0.91	0.79–1.05
BMI Centered					1	0.98–1.01
Exerciser						
Yes					Reference	
No					1.06	0.82–1.38
Former					1.08	0.72–1.63
Log of Income					1.13	0.95–1.34
Residence						
Province or City					1.04	0.50–2.17
County					0.98	0.65–1.47
Township					1	0.75–1.35
Village					Reference	
Education Level						
Jr. High or Less					Reference	
High School / Technical School					1.03	0.79–1.34
3 Year College					0.99	0.71–1.37
4 Year College or More					0.75	0.42–1.34
Medical Insurance Coverage					1.11	0.80–1.54
Past Live Birth					0.91	0.70–1.20
Pregnancy Was Planned					1.08	0.84–1.38
Family Gender Preference						
Prefer Boy					Reference	
Prefer Girl					1.34	0.70–2.57
No Preference					1.47	0.89–2.44

Model 1: IWGA, Unadjusted Logistic Regression; Model 2: IWGA Logistic Regression Adjusted For Imbalance; Model 3: IWGA Logistic Regression Adjusted For Imbalance and Potential IWGA Inputs; * *p* < 0.1, *** *p* < 0.01; γ Significant after a Holm-Bonferroni correction for the 4 planned group comparisons; ϕ Significant after a Holm-Bonferroni correction for all 6 possible group comparisons.

**Table 4:** Logistic Regression models with SGA and Macrosomia as dependent variables, Adjusted for Imbalance and Potential IWGA Inputs.

Variables	SGA		Macrosomia	
	Odds Ratio	95% CI	Odds Ratio	95% CI
Treatment Assignment				
Control Group	Reference		Reference	
CS Group	1.06	0.73–1.55	0.54 **	0.34–0.87
GHPP Group	0.91	0.62–1.35	0.66	0.42–1.03
All Texts Group	0.76	0.52–1.13	0.57 **	0.36–0.89
Gestational Week at Enrollment	1.01	0.99–1.03	1	0.97–1.02
Past Miscarriage	0.68 **	0.51–0.92	1.69 ***	1.19–2.40
Smoker	1.18	0.33–4.22	0.95	-
Husband Smoker	1.12	0.83–1.50	0.65 **	0.46–0.92
Health Compared to Before Pregnancy				
Better	1.11	0.58–2.13	0.18 *	0.03–1.16
The Same	Reference		Reference	
Worse	0.78	0.52–1.16	0.82	0.53–1.27
Not Sure	1.16	0.74–1.81	0.43 **	0.20–0.89
Maternal Gender Preference				
No Preference	Reference		Reference	
Prefer Boy	0.98	0.52–1.83	1.21	0.58–2.53
Prefer Girl	1.36	0.93–2.00	1.01	0.62–1.63
BMI Centered	0.98	0.96–1.00	1.02	0.99–1.04
Exerciser				
Yes, Current	Reference		Reference	
No	1	0.73–1.37	1.13	0.77–1.67
Former	0.81	0.47–1.38	1.51	0.87–2.61
Log of Income	1.16	0.89–1.50	1.07	0.86–1.34
Residence				
Province or City	1.03	0.41–2.56	1.1	0.37–3.29
County	0.72	0.41–1.25	1.37	0.79–2.37
Township	0.92	0.63–1.33	1.11	0.71–1.73
Village	Reference		Reference	
Education Level				
Jr. High or Less	Reference		Reference	
High School/Technical School	1.07	0.77–1.49	0.98	0.65–1.48
3 Year College	0.9	0.60–1.35	1.18	0.73–1.90
4 Year College or More	0.85	0.43–1.68	0.63	0.23–1.72
Medical Insurance Coverage	0.92	0.62–1.36	1.47	0.87–2.50
Previous Live Birth	0.77	0.54–1.08	1.18	0.80–1.73
Pregnancy Was Planned	1.13	0.83–1.54	0.98	0.68–1.42
Intentions	0.86 *	0.72–1.03	1.03	0.83–1.28
Family Gender Preference				
Prefer Boy	Reference		Reference	
Prefer Girl	0.84	0.39–1.81	2.94 *	0.93–9.30
No Preference	0.88	0.49–1.60	3.33 **	1.28–8.68

* *p* < 0.1, ** *p* < 0.05, *** *p* < 0.01.
